# Emerging Role and Therapeutic Implication of Wnt Signaling Pathways in Autoimmune Diseases

**DOI:** 10.1155/2016/9392132

**Published:** 2016-03-27

**Authors:** Juan Shi, Shuhong Chi, Jing Xue, Jiali Yang, Feng Li, Xiaoming Liu

**Affiliations:** ^1^Center of Laboratory Medicine, The General Hospital of Ningxia Medical University, Yinchuan 750004, China; ^2^Department of Rheumatology, The General Hospital of Ningxia Medical University, Yinchuan 750004, China; ^3^Key Laboratory of the Ministry of Education for the Conservation and Utilization of Special Biological Resources of Western China, Ningxia University, Yinchuan, Ningxia 750021, China; ^4^Institute of Human Stem Cell Research at the General Hospital of Ningxia Medical University, Yinchuan, Ningxia 750004, China

## Abstract

The Wnt signaling pathway plays a key role in many biological aspects, such as cellular proliferation, tissue regeneration, embryonic development, and other systemic effects. Under a physiological condition, it is tightly controlled at different layers and arrays, and a dysregulated activation of this signaling has been implicated into the pathogenesis of various human disorders, including autoimmune diseases. Despite the fact that therapeutic interventions are available for ameliorating disease manifestations, there is no curative therapy currently available for autoimmune disorders. Increasing lines of evidence have suggested a crucial role of Wnt signaling during the pathogenesis of many autoimmune diseases; in addition, some of microRNAs (miRNAs), a class of small, noncoding RNA molecules capable of transcriptionally regulating gene expression, have also recently been demonstrated to possess both physiological and pathological roles in autoimmune diseases by regulating the Wnt signaling pathway. This review summarizes currently our understanding of the pathogenic roles of Wnt signaling in several major autoimmune disorders and miRNAs, those targeting Wnt signaling in autoimmune diseases, with a focus on the implication of the Wnt signaling as potential biomarkers and therapeutic targets in immune diseases, as well as miRNA-mediated regulation of Wnt signaling activation in the development of autoimmune diseases.

## 1. Introduction

The evolutionary process of organisms leads to development of immune system enabling the body to identify “self-” and “nonself”-components to maintain an immune homeostasis, and a malfunction of immune system may cause abnormally low activity or over activity of the immune system. The low activity is a cause of immune deficiency diseases that decrease an ability to eliminate invaded pathogens, and the overwhelming activity leads the body to attack and damage its own tissues and cause autoimmune diseases (ADs).

An autoimmune disease (AD) can affect one or many different types of body tissues, which can be characterized by self-immune disorders involving chronic inflammation, causing multiple major organ damages and failures, as well as the accumulation of autoantibodies in genetically susceptible individuals. They can generally be further grouped into two categories: systemic autoimmune diseases such as systemic lupus erythematosus (SLE) and organ- or tissue-specific autoimmune diseases including rheumatoid arthritis (RA) and inflammatory bowel disease (IBD) [1]. There are more than 80 types of autoimmune diseases ranging in severity from mild to severe, depending on the degree and/or organ(s) affected. In addition, many ADs have similar symptoms, which lead to a difficulty in diagnosis of ADs. Though several ADs, such as RA, can be managed by suppressing chronic inflammations and preventing organ damages using agents including nonsteroidal anti-inflammatory drugs (NSAIDS), antimalarials, steroids, immunosuppressive agents, and biological therapies, the treatment for autoimmune diseases mainly focuses on relieving symptoms, since there is no curative therapy currently available, and the adverse effects of long-term administration of these therapeutic agents limit their uses on patients with multiple organ complications [2]. Even worse, a large number of ADs are currently lacking an effective and successful specific therapy, in part owing to the incomplete understanding of the underlying mechanisms of signaling regulation of these ADs [3, 4].

The Wnt signaling has been demonstrated to play crucial roles in several biological aspects, including cellular proliferation, embryonic development, tissue homeostasis, development of immune system, and other systemic effects [5]. In addition to its dispensable roles in the development of T cells and the immune system, mounting evidence has recently suggested that this signaling pathway is involved in the pathogenesis of many types of autoimmune diseases, such as RA, SLE, and ankylosing spondylitis (AS) [6–12]. Recently, emerging roles of microRNAs (miRNAs), a class of small, noncoding RNAs capable of transcriptionally regulating target gene expression in the autoimmune diseases, have also been recognized. Some of these miRNAs have been implicated in the pathogenesis of ADs, through mechanisms by targeting Wnt signaling pathways. Hence, we summarized our current understanding in the emerging roles of Wnt signaling in autoimmune diseases and miRNAs, those targeting Wnt signaling components in ADs, with a focus on Wnt family members and miRNAs targeting this signaling as potential molecular targets for the diagnosis and treatment of autoimmune diseases.

## 2. The Wnt Signaling Pathways

The Wnt signaling is an ancient and evolutionarily conserved pathway identified in metazoan animals, which has been well characterized to play pivotal roles in cell fate determination, cell migration, cell polarity, neural patterning, and organogenesis during embryonic development, stem cell maintenance, and tissue homeostasis during physiological condition and tissue repair following injuries [5, 13, 14]. A dysregulation of Wnt signaling has been implicated in the pathogenesis of many disease types, including cancers and ADs [15–19]. To date, 19 human Wnt genes that encode respective Wnt proteins have been identified in humans [13]. Wnt proteins can bind to the seven transmembrane spanning frizzled (Fzd) receptors that are also able to complex with coreceptors such as the low-density lipoprotein-related receptor (LRP) 5 or 6 [13]. However, Wnt proteins are not restricted to specific Fzd receptors; an interaction of Wnt/Fzd may activate several different pathways. Wnt signaling pathways can be thus further characterized by a “canonical pathway” and several “noncanonical pathways,” including the Wnt/*β*-catenin, planer cell polarity (PCP), c-Jun N-terminal protein kinases (JNK), protein kinase C/calcium (PCK/Ca^2+^), receptor-like tyrosine kinase (RYK), and receptor tyrosine kinase-like orphan receptor (Ror) pathways [5, 13, 20]. Among these, the Wnt/*β*-catenin signaling pathway is the most investigated and the best characterized Wnt signaling pathway.

**Figure 1:**
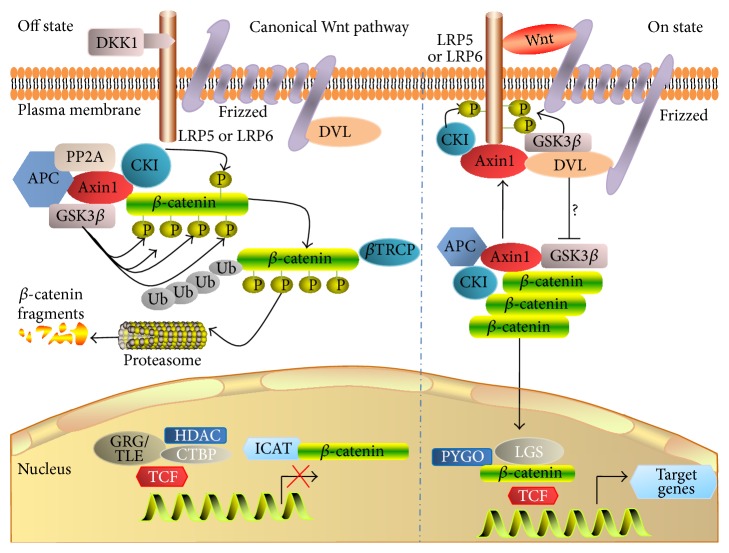
Canonical Wnt signaling pathway (*β*-catenin-dependent Wnt signaling pathway). In the absence of Wnt ligand(s), cytoplasmic *β*-catenin is targeted for phosphorylation, by a multiprotein complex comprising Axin, adenomatous polyposis coli (APC), the glycogen synthase kinase 3*β* (GSK3*β*), and casein kinase 1*α* (CK1*α*). The phosphorylated form of *β*-catenin is recognized by an E3 ubiquitin ligase (*β*-TrCP) and then targeted to proteasomal degradation, resulting in low cytosolic levels (left panel); in the presence of Wnt ligand(s), Wnt ligand binds to the Fzd and LRP receptors, and this binding triggers the signaling and activates the Dvl; the activation of Dvl inhibits the GSK-3*β* and results in destructing the multiprotein complex which stabilizes and leads to the intracellular accumulation of *β*-catenin in the cytoplasm; accordingly the active *β*-catenin translocates to the nucleus, where it acts as a transcriptional coactivator with TCF/LEF to activate Wnt-responsive target genes (right panel).

The Wnt/*β*-catenin pathway also is referred to as the “canonical Wnt pathway,” which is characterized by Wnt binding to its coreceptor complex that is constituted by the LRP-5 or LRP6, and a member of the ten Fzd family of proteins (Figure 1) [21]. In a steady state without a Wnt signaling ligand, the cytosolic *β*-catenin is phosphorylated by the complex consisting of glycogen synthase kinase 3*β* (GSK3*β*), casein kinase I (CKI), Axin, and adenomatous polyposis coli (APC) [22]. In this context, Axin is a scaffold that favors the union of the GSK3*β* to cytosolic *β*-catenin to phosphorylate *β*-catenin, and APC mediates phosphorylated *β*-catenin binding to the ubiquitin-mediated proteolysis pathway. In the presence of Wnt protein ligand, Wnt binds to its coreceptor complex and activates Wnt signaling by recruiting the cytosolic disheveled (Dvl) proteins, which in turn blocks the *β*-catenin degradation and sequentially leads to the accumulation of *β*-catenin in cytoplasm and translocation of *β*-catenin into nucleus and binds to transcriptional factors TCF/LEF to initiate the expression of Wnt target genes [13].

**Figure 2:**
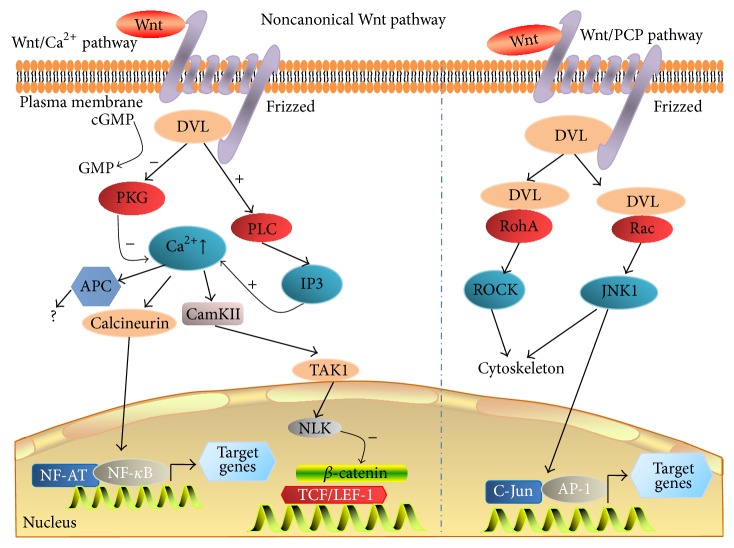
Noncanonical Wnt signaling pathway (*β*-catenin-independent Wnt signaling pathway). Noncanonical Wnt ligand (such as the Wnt5a, a typical noncanonical Wnt) binds to its receptor (Fzd) and coreceptor (Ror1/2) and triggers the noncanonical signaling cascades, which includes the Wnt/Calcium (Ca^2+^) and Wnt/planar cell polarity (PCP) pathways. In the Wnt/Ca^2+^ pathway (left panel), Wnt protein binds to Fzd and Ror2 receptor and leads to activate G proteins, resulting in enhancing the intracellular calcium levels, or decreases cGMP; the calcium/calmodulin-dependent protein kinase II (CaMKII) or protein kinase C (PKC) was then activated. In the Wnt/PCP pathway (right panel), Wnt proteins bind to Fzd receptors on the cell surface followed by activating Rho/Rac small GTPase and Jun N-terminal kinase (JNK) to assist with cytoskeletal organization and gene expression.

A Wnt signaling pathway that is independent *β*-catenin-TCF/LEF is classified into the “noncanonical signaling pathway” (Figure 2), which may regulate both transcriptional and nontranscriptional responses in the cells [23]. The planar cell polarity (PCP) and the Wnt/Ca^2+^ pathways are two of the best characterized *β*-catenin-independent noncanonical Wnt pathways [24]. The PCP pathway is characterized by a Dvl-driven sorting of cellular components to either the proximal or distal regions of the cell and directs it within the tissue [25], within which the Fzd receptor activates a cascade that has the small GTPases RAC1 and Ras homolog gene family, member A (RHOA), and JNK as downstream effectors that direct asymmetrically cytoskeletal organization and coordinated polarization of cells within the plane of epithelial sheets by controlling target gene expression [26]. In the PKC/Ca^2+^ pathway, a Wnt ligand binding to Fzd receptor triggers the activation of heterotrimeric G proteins, which is in turn able to activate phospholipase C (PLC) and PKC and sequentially leads to the release of intracellular Ca^2+^. The evoked Ca^2+^ concentration activates the phosphatase calcineurin, which leads to dephosphorylation of transcription factor, nuclear factor of activated T cells (NFAT) to regulate the transcription of genes controlling cell fate and cell migration [24].

Interestingly, Wnt signaling can be regulated by both of intracellular proteins which influence signal transduction and extracellular antagonists such as Wise (Sostdc1), secreted frizzled-related protein (SFRP), the Wnt inhibitory factor 1 (WIF-1), Cerberus, and the dickkopf (DKK) family of secreted proteins [27]. Among them, the DKK family of Wnt antagonists has recently spurred increased interests. The DKK family comprises four members of proteins, DKK1, DKK2, DKK3, and DKK4, which are synthesized as precursor proteins activated by a proteolytic cleavage [28]. The DKK1 and DKK3 are the most studied members of this family, which can inhibit the Wnt signaling by binding to LRP5/6 and then degrading the coreceptor, and thus have been considered as potential targets in diseases with an aberrant Wnt signaling activity [29, 30].

To date, biological roles of Wnt signaling in T cell development have been well established [31], and its roles in immune regulation have also recently gained an increased attention [32]. Moreover, accumulating evidences have indicated that the Wnt signaling has clinical implications in the pathogenesis of many types of autoimmune diseases, including RA, SLE, and AS [6, 8, 9, 11, 17, 18, 33–35].

## 3. Regulatory Roles of Wnt Signaling in Immune Cells

Hematopoietic stem cells (HSCs) are capable of differentiating into hematopoietic progenitor cells (HPCs), which can further differentiate into immune cells, such as T cells, B cells, NK cells, and macrophages. There are many lines of evidence which have demonstrated that Wnt signaling plays a key role in the maintenance, proliferation, differentiation, and self-renewal of HSCs [36]. Wnt ligand proteins and receptors can be produced not only by HSCs and primitive progenitors, but also by the cells constituting the surrounding microenvironment in both adult and fetal hematopoietic organs of mice and humans [37]. For example, indeed, Fzd6, a noncanonical Wnt receptor, could promote HPC expansion and multilineage hematopoietic recovery after the transplantation [38].

Apart from its regulatory roles in HSCs, the importance of Wnt signaling in the T cell development has also been well established [37]. In this context, the Wnt signaling provides crucial proliferative signals to immature T cells, which is supported by studies in mice deficient for the Wnt-responsive transcription factors TCF1 and LEF1, in which the development of T cells and B cells was, respectively, defective [37]. In addition, a canonical Wnt/*β*-catenin/T cell factor (TCF) pathway has been shown to regulate T cell differentiation in both the thymus and in peripheral lymphoid tissues, and a dysfunction of this pathway at any stage of T cell differentiation could lead to severe autoimmunity or immune deficiency [39]. For example, a transcriptome analysis of the CD4 T cells in rheumatoid revealed an aberrant regulation of signal transducer and activator of transcription 3 (STAT3) and Wnt signaling pathways [40]. Moreover, regulatory T (Treg) cells have an important role in regulation of immunity. The expression of a stable form of *β*-catenin could lead to a remarked enhancement of survival of CD4^+^/CD25^+^ Treg cells* in vitro*, suggesting that a stabilization of *β*-catenin has an impact on the prevention of inflammatory disease by enhancing the survival of existing Treg cells and keeping precursors of T effector cells unresponsiveness [41]. Inconsistent with a steady state, an activation of Wnt signaling in an inflammatory condition could repress Treg cell function, which in turn allowed triggering of an immune response. However, if the process was uncontrolled, that could lead to the development of autoimmune responses [42].

B cells are generally recognized for their potential to mediate humoral immunity by producing different antibody isotypes and being involved in opsonization and complement fixation [79]. The Wnt signaling is critical for differentiation of HSCs into B cells with normal function. Unlike the fact that there is a compelling study in Wnt-regulated T cell development, studies in functions of Wnt signaling in B cells are currently limited, although there are several lines of direct evidence of aberrant Wnt signals in B cells which were related to autoimmunity [59, 80, 81]. For example, the Wnt canonical pathway could induce B-1 cell survival and proliferation by stimulating the expression of Wnt target genes of c-Myc and cyclin D1 [59].

Dendritic cells (DCs) are antigen-presenting cells (APCs) that play an important role in regulating immune responses and balancing the innate and adaptive immune responses. However, factors in tissue microenvironments and the signaling networks critical for programming DCs to control the chronic inflammation and promote the immune tolerance are unknown. To date, a growing body of evidence suggests that Wnt signaling pathways are pivotal pieces of the immune balance and able to directly target DCs [32]. For instance, an activation of Wnt/*β*-catenin signaling in DCs was found to be critical for promoting tolerance and limiting neuroinflammation in mice with an exacerbated experimental autoimmune encephalomyelitis, in which the expression of LRP5/6 or *β*-catenin was downregulated in DCs [82]. Noteworthy, such a decreased LRP5/6-*β*-catenin-mediated signaling led to an increased Th1/Th17 cell differentiation but reduced Treg cell response, owing to the increased production of proinflammatory cytokines and reduction of anti-inflammatory cytokines such as IL-10 and IL-27 [82]. This finding suggests that an activation of Wnt/*β*-catenin signaling could delay experimental autoimmune encephalomyelitis onset and diminished its CNS pathology [82].

Wnt5a is a noncanonical Wnt signaling ligand, which has been demonstrated as key player in the pathogenesis of atherosclerosis and other inflammatory disorders [83]. Consistently, Valencia et al. showed that an increased concentration of Wnt5a in monocytes promoted their differentiation into unconventional DCs with tolerogenic features in the inflammatory state and sepsis, in which unconventional DCs produced scant amounts of IL-12p70 and TNF-*α* and a reduction of IL-10. As a consequence, these Wnt5a-DCs have a reduced capacity to induce Th1 responses that promote IL-10 secretion by CD4^+^ T cells [84]. A later study further revealed that activated DCs which could promote naïve CD4^+^ T cells turn to FoxP3^+^ Tregs in an indoleamine 2,3-dioxygenase-1- (IDO-) dependent manner and induce an immune tolerance [85]. For example, both canonical and noncanonical Wnt ligands can directly stimulate murine intestinal surface DCs to produce anti-inflammatory cytokines, and canonical Wnt signaling ligand Wnt3a can activate *β*-catenin signaling and preferentially induces DC to produce TGF-*β* and VEGF, but Wnt5a activates noncanonical signaling and induces IL-10 secretion. Hence, Wnt family members regulate DC functions that may contribute to the balance between tolerance and inflammation when intestine is exposed to microbe and food in a mouse model of inflammatory bowel disease [86, 87]. These studies clearly imply that the Wnt signaling plays a regulatory role in the homeostasis of immune system.

Given the fact of hyperactivation of immune cells in ADs [88, 89], an aberrant Wnt signaling activation thus has been suggested to contribute to the pathogenesis of ADs. Despite that fact that there are few evidences which directly support the view that the Wnt singling has an impact on autoimmunity by regulating HSCs, the autoimmunity attributed by a dysfunction of HSCs is related to a dysregulated Wnt signaling activity. For instance, acetylsalicylic acid (ASA) which activated stem cells from exfoliated deciduous teeth (SHED) could significantly improve SHED-mediated osteogenic differentiation and immunomodulation, increase SHED-mediated T cell apoptosis, and ameliorate disease phenotypes in dextran sodium sulfate-induced colitis mice, by upregulating the telomerase reverse transcriptase (TERT)/Wnt/*β*-catenin signaling cascade [90]. Currently, a compelling of study has demonstrated involvements of Wnt signaling pathways in the pathogenesis of ADs (Table 1). Among these, the pathogenic roles of Wnt pathways in several major ADs including RA, AS, and SEL have gained an increasing interest.

## 4. Wnt Signaling as Potential Biomarkers and Therapeutic Targets in Autoimmune Diseases

Currently, increasing numbers of studies have demonstrated involvements of Wnt signaling pathways in the pathogenesis of ADs (Table 1). Among these, the pathogenic roles of Wnt pathways in several ADs including RA, AS, and SEL have gained an increasing interest.

### 4.1. Wnt Signaling in Rheumatoid Arthritis (RA)

RA is a chronic, debilitating autoimmune disease that results in inflammation and structural destruction of the joints, which is probably the results of a combination of genetic and environmental factors, and a dysregulation of signaling networks [91]. With respect to signaling networks, Wnt signaling has been implicated in the etiology of RA, although the exact nature of their involvement remains unclear [18, 34, 35].

In patients with RA, Wnt7b is a member of the Wnt gene family most closely linked to RA, which was significantly upregulated in RA synovium, along with an increased production of inflammatory mediators TNF-*α*, IL-1*β*, and IL6. Interestingly, an elevated expression of TNF-*α*, IL-1*β*, and IL6 was also found in Wnt7b-transfected normal synovial cells;* vice versa*, an increased level of the inflammatory mediators in turn induced the Wnt/Fzd expression, suggesting a potential involvement of Wnt signaling in the pathobiology of RA [48]. Additionally, higher levels of Wnt ligands, Fzd receptors, and Wnt inducible signaling pathway proteins (WISP) were observed in the synovium of RA patients compared to those in patients without RA [46, 48]. Interestingly, an activation of the Wnt/*β*-catenin signaling in chondrocytes induces cartilage matrix degradation similar to that which occurs in osteoarthritis and RA, whereas the blockade of Wnt signaling facilitates bone erosion and might contribute to the catabolic model in the bone remodeling observed in RA patients, in which the canonical Wnt pathway was able to regulate the expression of fibronectin and metalloproteinase [7, 18].

Functionally, an aberrant expression of Wnt signaling components has been implicated in promotion of cell growth and differentiation [46].* In vitro* studies have shown that fibroblast-like synoviocytes (FLS) from patients persistently expressed high levels of both Wnt5a and Fzd5, suggesting that an activation of noncanonical Wnt5a/Fzd5 signaling may contribute to the activated state of FLS in RA, despite the fact that the constitutive activation of Wnt/Fzd signaling in RA was independent of an inflammatory environment [46, 92]. Thus, receptor antagonists of Fzd5 have been considered for the treatment of refractory synovitis [47]. Of note, Miao et al. recently identified that an increased methyl-CpG-binding protein 2 (MeCP2) expression led to a reduced expression of secreted frizzled-related protein 4 (SFRP4), a negative regulator of Wnt signaling in FLS of arthritic rat model; in contrast, an addition of 5-Aza-2′-deoxycytidine (5-azadC), an inhibitor of DNA methyltransferase (DNMT), could induce the SFRP4 expression. Recently, by using loss and gain of function experiments* in vitro*, Miao et al. identified that the methyl-CpG-binding protein (MeCP2) had a function on regulation frizzled-related protein 4 (SFRP4) expression in RA through a mechanism of epigenetic modification of SFRP4 gene, which could be used as a diagnostic marker and prognostic indicators of RA [34, 35]. Mechanistically, a downregulation of MeCP2 resulted in the dissociation of interaction of Wnt protein and its Fzd receptor by SFRP4, and the formation of complex of *β*-catenin, GSK3*β*, Axin, CK1*α*, and APC sequentially led to the *β*-catenin phosphorylation by GSK3*β*. The phosphorylated *β*-catenin then was degraded by ubiquitination [34]. In addition, the canonical Wnt/*β*-catenin signaling also plays a central role in the bone development and homeostasis in adulthood, and a dysregulation of this signaling is associated with bone pathologies [93]. In this context, dickkopf-1 (DKK1), a soluble inhibitor of canonical Wnt signaling, which is required for embryonic head development and regulates Wnt signaling by binding to the Wnt coreceptor LRP5, which has been implicated in causes of erosive arthritis, and several preclinical studies have shown that a neutralizing DKK1 and/or enhancing Wnt/*β*-catenin signaling may be an effective therapeutic option in treatment of bone pathologies [45, 94, 95].

With respect to therapeutic stand point of RA, an inhibition of synovitis has been focused on this disease, since the imbalance of the osteoblast-osteoclast axis driven by inflammatory processes is a hallmark of RA pathogenesis, which leads to an elevated bone resorption by osteoclasts [96]. However, such a treatment strategy may be not adequate for damaged bone repairing. Therefore, a regimen with a combination of such treatments and anti-inflammatory therapies may be able to stabilize and repair damaged joints and have the potential to be valuable additions to the armory of RA treatments [44]. The Wnt signaling has been implicated in the differentiation of osteoblasts from mesenchymal lineage precursors, and the receptor activator of nuclear factor *κ*B ligand (RANKL) pathway has also been suggested to be involved in osteoclast formation by acting on myeloid progenitor cells [90], along with the fact that Wnt inhibitors including the DKK1 and sclerostin may play important roles in osteoclast dysregulation in RA [95], an inhibition of the RANKL pathway, or blockade of DKK1 and sclerostin and thus can serve to restore the osteoblast-osteoclast balance and repair bone erosion in RA joints.

### 4.2. Wnt Signaling in Ankylosing Spondylitis (AS)

AS is an inflammatory disease that affects the axial skeleton and the peripheral joints [97]. Although the mechanisms of AS pathogenesis are not currently fully understood, the Wnt signaling has been recently implicated as a key pathway during the development of AS, owing to its involvement in bone morphogenesis and homeostasis by inducing mesenchymal cells differentiation into the osteoblast lineages [17], which may play an essential role in the anabolic pattern of joint remodeling observed in AS and osteoarthritis [7]. Indeed, aberrant TNF has been suggested to contribute to induce Wnt inhibitors evoking expression of DKK-1 and sclerostin in AS [98]. A neutralization of DKK-1 with antibodies exhibited a reversed phenotype of erosions in several inflammatory arthritis murine models and altered the phenotype from bony erosion to proliferation [98]. Furthermore, the blockade of DKK1 with this antibody could promote the fusion of the sacroiliac joints in TNF-engineered AS mouse model [99]. Mechanistically, the TNF-induced release of DKK1 might be able to inhibit Wnt signaling, which in turn diminished osteoprotegerin (OPG) expression and osteoblastogenesis and increased osteoclast activity and erosion [98]. This notion was in accordance with the finding of a decreased serum DKK1 in RA patients but not in the AS patients treated with TNK inhibitors [98]. Pathogenically, the site of inflammation of AS was found to incorporate first a relative adipose accumulation and then bone overgrowth in the form of a syndesmophyte, and an increase of the fat signal in MRI imaging of the vertebrae after inflammation subsides could be observed from treatment with a TNF inhibitor [100]. In this regard, Wnt signaling typically is capable of suppressing adipocyte adipogenesis [101].

In addition, both sclerostin and DKK1 have been suggested as biomarkers for disease activity in AS [102], and patients in the German Spondyloarthritis Inception Cohort (GESPIC) were higher risk of developing a syndesmophyte if they had lower levels of circulating functional DKK1 as determined in terms of coating the plate with purified LRP6 but not a capture antibody [103]. Interestingly, the amount of DKK1 protein was found not to be consistently correlated with its capacity of binding to the LRP coreceptor in sera of AS patients, in which the DKK1 in the sera was less able to suppress *β*-catenin translocation to the nucleus than control sera, implying that the DKK1 might be dysfunctional in AS patients [50].

### 4.3. Wnt Signaling in Systemic Lupus Erythematosus (SLE)

SLE is an autoimmune disease with multisystem, multiorgan injury, and productions of a variety of autoantibodies, which is caused by interactions of genetic and environmental factors and imbalance of immune system such as the imbalance of T cells [2]. An aberrant expression of canonical Wnt signaling related genes HIG2, TCF7, KHSRP, WWP1, SMAD3, TLK2, AES, CCNI, and PIM2 was observed in the peripheral blood CD4^+^ T cells in patients with SLE [104]. These genes have been demonstrated to play an important role in the regulation of T cell proliferation and differentiation [39]. A whole genome analysis further revealed that DKK4, an important inhibitor of Wnt/*β*-catenin pathway, was abnormal in Chinese Han patients with SLE relative to healthy control cohorts [105]. Consistently, another DKK family member DKK1 was found to be significantly higher in the sera of SLE patients in comparison with controls, which was positively correlated with their bone erosion, suggesting that DKK1 might be a valuable biomarker for SLE [106].

The lupus nephritis (LN) is an important part of clinical manifestation of SLE; the LN MRL/lpr mice exhibited a phenotype with an enhanced Wnt/*β*-catenin activity, accompanied by an increased level of DKK1 in the renal tissues and sera and an increased frequency of apoptotic cells of the renal tubular and renal interstitial tissues [106]. Such an elevated Wnt signaling activity was further evidenced in human renal tissues of patients with LN by accessing *β*-catenin at both transcriptional and translational levels using assays including immunohistochemistry staining, qRT-PCR, and western blotting, suggesting that a dysregulated Wnt/*β*-catenin signaling was related to the pathogenesis of LN and might play a role in the renal fibrosis [11]. This notion may be in part supported by findings of low circulating complement C1q and abundant C1q deposition in LN renal tissues. Indeed, low levels of circulating complements, including C1q, C3, and C4, are reported in SLE patients. A recent study demonstrated that C1q was able to activate the canonical Wnt signaling and promote aging related gene expression [107]. In SLE and LN patients, circulating complement C1q level was significantly reduced, which was correlated with SLE diseases activity [108]. Intriguingly, in contrast to low level of C1q seen in sera, an abundant complement C1q deposition was found in the kidneys of LN patients [108]. As a consequence, the deposition of C1q might be able to direct the circulating anti-C1q antibodies that abundantly existed in sera to attack the renal tissues, which in turn resulted in a deposition of immune complexes in the glomeruli and renal inflammation and fibrosis [108]. Together with a capacity of C1q to activate Wnt *β*-catenin signaling [107] and evidences of hyperactivated canonical Wnt signaling during lupus development in mice [8] and in renal biopsy of LN patients [11], these findings clearly imply that the deposition of C1q may activate Wnt/*β*-catenin signaling in renal, which has a significant implication in the pathogenesis of LN [11].

The balance of Th17 and Treg cells is also an important implication in the pathogenesis and excessive immune responses of SLE [109]. Accumulating evidences have suggested that Wnt ligand proteins and signaling effectors play a critical role in the regulation of Th17 cell differentiation and function [110]. In this context, the Wnt signaling pathway inhibitor SFRP1 showed an ability to increase Smad2/3 phosphorylation in CD4^+^ T cells in response to TGF-*β* stimulation and sequentially induce Th17 cell differentiation [111]. In addition, the Wnt signaling can also regulate Treg cells by regulating the transcriptional activator protein osteopontin, nuclear receptor subfamily 4, group A, member 2 (NR4A2), and a target protein of Wnt/*β*-catenin signaling pathway. The NR4A2 can transcriptionally activate inflammatory cytokines such as TNF-*α* and IL-1*β* but also activate Forkhead box P3 (FoxP3) [112, 113]. The FoxP3 is a crucial transcription factor in Treg cell differentiation and maintenance and cellular functionality [42].

With regard to the therapeutic implication, recent studies have shown that allogeneic but not syngeneic bone marrow- (BM-) mesenchymal stem cell transplantation (MSCT) appears to be effective in SLE patients and lupus-prone mice [114]. These findings indicate that the abnormalities of BM-MSCs may contribute to the pathogenesis of SLE. In this regard, BM-MSCs from SLE patients exhibited characteristics of senescence [115], which might be attributed to a dysregulated Wnt signaling pathway, as demonstrated in other stem cell populations [116]. Indeed, such notion was supported by a recent proof-of-concept study, in which the investigators examined the impact of Wnt/*β*-catenin signaling on the senescence of BM-MSCs from SLE patients [68]. Intriguingly, both the canonical Wnt signaling and p53/p21 signaling were hyperactivated in senescent SLE BM-MSCs. However, SLE BM-MSCs exposed to Wnt pathway inhibitor DKK1 or transduced *β*-catenin siRNA exhibited alleviated features of cell senescence, along with a reduced expression of p53 and p21, indicating that Wnt/*β*-catenin signaling may be a potential target for allogeneic BM-MSC-mediated cell therapy for SLE [68].

### 4.4. Wnt Signaling in Systemic Sclerosis (SSc)

SSc is a rare autoimmune disease with high mortality, which is characterized by immune hyperactivation along with vascular damage and an excessive accumulation of extracellular matrix proteins in the skin and internal organs. The antifibrotic therapy is an available treatment option for fibrotic manifestations of SSc in clinical settings, whose effects remain borderline, despite the fact that significant progresses have been made by the identification of a large number of cellular and molecular key players in the pathogenesis of fibrotic disease manifestations in the past decade [54].

An elevated Wnt signaling activity with increased expression of Wnt signaling components Wnt1, Wnt10b, Fzd2, nuclear *β*-catenin, and LEF-1 proteins and decreased expression of DKK2 and WIF-1 in skin fibroblasts was observed in SSc patients, which were positively correlated with skin fibrosis [9, 53, 55, 117]. Mice with fibroblast-specific dominant active *β*-catenin rapidly developed fibrosis within 2 weeks with dermal thickening, accumulation of collagen, and differentiation of fibroblasts into myofibroblasts. Conversely, fibroblast-specific deletion of *β*-catenin significantly reduced bleomycin-induced dermal fibrosis [9]. However, activation of Wnt/*β*-catenin signaling by inhibition of mouse fibroblasts GSK3*β* using siRNA led to a decreased collagen synthesis in fibroblasts and alleviated skin thickening in mice [118]. Intriguingly, WIF-1 deficiency in fibroblasts of SSc patients or knocking down WIF-1 in normal fibroblasts was correlated with an increased abundance of *β*-catenin and the production of collagen [55]. Mechanistically, the DNA damage checkpoint kinase ataxia telangiectasia mutated (ATM) could induce WIF-1 silencing via the phosphorylation of the transcription factor c-Jun, which in turn activated the expression of the gene encoding activating transcription factor 3 (ATF3). The ATF3 and c-Jun were recruited together with histone deacetylase 3 (HDAC3) to the WIF-1 promoter for repressing WIF-1 expression. In contrast, the of WIF-1 expression in cultured patient SSc cells could be restored by preventing the accumulation of reactive oxygen species (ROS) or inhibiting the activation of ATM, c-Jun, or HDACs. Therefore, together with the fact that HDAC inhibitor trichostatin A could prevent WIF-1 decrease, *β*-catenin activation, and collagen accumulation in an experimental fibrosis model, these studies suggested that the activation of Wnt signaling might contribute to SSc fibrosis [55].

In addition, an* in vitro* study further demonstrated that Wnt3a could induce *β*-catenin activation to stimulate fibroblast proliferation and migration, collagen gel contraction, and myofibroblast differentiation, along with an enhanced expression of profibrotic genes. In contrast, Wnt3a showed an ability to repress adipogenesis but promote myofibroblast differentiation in explanted subcutaneous preadipocytes [53]. These studies clearly evidenced that the canonical Wnt signaling was a key player of fibroblast activation and tissue fibrosis in SSc. Therefore, approaches and agents targeting Wnt signaling pathways, including Tankyrase inhibitors, porcupine inhibitors, and antagonists of cofactor recruitment to *β*-catenin, have been interrogated for their potential of antifibrotic effect and SSc treatment in animal models and pulmonary fibrosis models with [119, 120].

### 4.5. Wnt Signaling in Inflammatory Bowel Disease (IBD)

IBD is an idiopathic disease caused by a dysregulated immune response to host intestinal microflora, which involves a group of chronic inflammation of the colon and small intestine. IBD primarily includes ulcerative colitis (UC), which causes long-lasting inflammation and ulcers in colon and rectum, and Crohn disease (CD), which can cause inflammation in any segment of the gastrointestinal tract. IBD has a genetic predisposition, and IBD patients are more prone to develop intestinal carcinomas [121].

To date, an involvement of Wnt signaling in IBD pathogenesis has been broadly recognized [122]. Previous studies have revealed a hyperactivated Wnt signaling with an aberrant expression of key components of Wnt signaling cascade, including Wnt2, Wnt5a, Wnt5b, Fzd2, Fzd4, Fzd6, LRP6, Dvl, DKK1, and SFRP1 in both UC and CD tissues [123–125]. Wnt-specific mRNA microarray analysis of Wnt pathway-related gene expression in human UC demonstrated that the expression of Wnt2b, Wnt3a, Wnt5b, Wnt6, Wnt7, Wnt9, Wnt11, Fzd3, Fzd4, DKK4, and Dvl2 was significantly increased, but expression of Fzd1 and Fzd5 was dramatically decreased in UC as compared to non-IBD patients [126]. IHC staining also revealed that the expression of *β*-catenin and Wnt-associated cancer genes E-cadherin, cyclin D1, and c-myc expressions was unregulated in human UC tissues [127].

CD can be characterized a chronic mucosal inflammation with two pertinent features: a specific decrease of Paneth cell-produced alpha-defensins and the presence of mucosal-adherent bacteria [33]. Wnt signaling has long been recognized as a key pathway in small intestinal stem cell maintenance and Paneth cell differentiation [128]. A decrease of Paneth cell alpha-defensins has been reported in CD patients, which is a primary factor in disease pathogenesis partially owing to an impaired Wnt signaling caused by dysfunction of TCF4 and LRP6 [64]. Recently, Beisner et al. demonstrated that the barrier dysfunction observed in the disease might be attributed to a reduced expression of the TCF1 in patients, in whom the intestinal TCF1-mediated Wnt signaling was found to be disturbed [65]. These findings indicated that the dysfunction of Wnt signaling pathway and Paneth cell biology is pathophysiological hallmarks CD and may be potential targets for new therapeutic approaches for CD [33].

With respect to Wnt signaling in UC, Cosín-Roger et al. found that M2 but not M1 macrophages were responsible for activation of Wnt signaling and decrease enterocyte differentiation in cocultured epithelial cells, which was in agreement with findings in the mucosa of UC patients, in whom an increased number of M2 macrophages were associated with the disease chronicity, an activation of epithelial Wnt signaling, and a decreased extent of enterocyte differentiation [129]. Similarly, Sato et al. most recently identified a Wnt5a/Ror2 signaling axis in promoting the signaling circuit of IL12 and IFN-*γ* in UC by using Wnt5a and Ror2-deficient mice [130].

Mechanistically, the Wnt signaling can mediate mucosal repair coordinated by M2 macrophages [129]. In this regard, the signal transducer and activator of transcription 6 (STAT6) were essential for M2 macrophages to promote mucosal repair through activation of the Wnt signaling pathway in 2,4,6-trinitrobenzenesulfonic acid- (TNBSA-) induced IBD mice [131]. Since genetic mutations of Wnt signaling molecules, such as APC, are rare in IBD and IBD-associated neoplasia, epigenetic alterations are major parts contributing to the dysregulated Wnt signaling activity in IBD. Indeed, methylation analysis of IBD tissues showed that methylation in ten Wnt signaling pathway genes, including APC1A, APC2, SFRP1, SFRP2, SFRP4, SFRP5, DKK1, DKK3, WIF-1, and LKB1, was frequent in IBD and IBD-associated neoplasia, which were associated with the progression of the disease, suggesting that Wnt signaling components may serve as biomarkers for IBD and IBD-associated neoplasia [132, 133]. With a therapeutic prospect, interestingly, a recent genome-wide expression profiling analysis revealed that the intestinal epithelial canonical Wnt signaling was activated but the noncanonical Wnt pathways were suppressed in IBD, and a transplantation of mesenchymal stem cells could suppress the canonical pathway and induce noncanonical signaling, which resulted in an inhibition of inflammation in an IBD rat model [134].

### 4.6. Wnt Signaling in Other Immune Diseases

An increasing number of evidences have also uncovered an involvement of Wnt signaling in the pathogenesis of many other types of ADs (Table 1) [1]. Below are some examples.

Alopecia areata (AA) is an autoimmune disease manifested hair loss, which is caused by a cell-mediated immune attack of the cycling hair follicle [57]. Impaired Wnt/*β*-catenin has been shown to stagnate anagen initiation and disable stem cells to differentiation to hair keratinocytes rather than epidermal cells instead [135, 136]. The inhibited Wnt/*β*-catenin activity may be attributed to an upregulation of canonical Wnt signaling inhibitors and activation of noncanonical Wnt pathway, leading to an UDP-mediated *β*-catenin degradation [5]. These findings are in line with the evidence of a decreased AA lesional skin with Wnt10b activated-canonical Wnt signaling [137] and an increased stemness of hair follicle stem cells induced by a vitamin A-enhanced Wnt signaling to be activated [57].

Type 1 diabetes mellitus (T1DM) is believed to be an autoimmune disease where the immune system attacks pancreatic *β*-cells in islets and abolish endogenous insulin production [138]. An enhanced canonical Wnt signaling by inhibiting GSK3*β* showed a potential for the regeneration of *β*-cell function and mass in patients with diabetes [70]. In this regard, both rat INS-1E insulinoma cells and isolated rat islets treated with GSK3*β* small molecule inhibitor or siRNA showed an increased proliferative capacity and resistance to high concentrations of glucose and the saturated fatty acid palmitate [70]. In line with this finding, activating Wnt/*β*-catenin signaling by overexpression of Wnt3a could promote porcine pancreatic stem cells (PSCs) proliferation and enhance the tolerance of cells to ROS-induced mitochondria injury and cell apoptosis [139]. These studies suggest that impaired Wnt pathway may contribute the T1MD pathogenesis and an activation of canonical Wnt signaling may have practical applications in beta cell regenerative therapies for T1MD.

Psoriasis is a disease characterized by chronic inflammation and altered differentiation and hyperproliferation of keratinocytes [140]. Wnt5a has been reported to be upregulated in lesional psoriatic skin as determined by gene expression and was shown to synergize with type 1 IFNs [67, 141]. Global gene expression profiling analysis of biopsies of human psoriatic skins revealed significantly upregulated Wnt5a transcripts and proteins along with an increased expression of Fzd2 and Fzd5 but decreased WIF-1 expression. Interestingly, the expression of Wnt5a could be induced by IL1*α*, TNF-*α*, IFN-*γ*, and TGF-*α* in cultured keratinocytes. Of interest, a reduced expression of Axin2 and lack of nuclear *β*-catenin suggested a suppression of canonical Wnt signaling in lesional skins, implying that the canonical Wnt signaling was shifted toward noncanonical pathways by activation Wnt5a-mediated signaling and impaired homeostatic inhibition of Wnt/*β*-catenin signaling by WIF-1 and DKK in psoriasis [67].

## 5. MicroRNAs Targeting Wnt Signaling Pathway in Autoimmune Diseases

MicroRNAs (miRNAs) are a class of noncoding, small RNA molecules found in both prokaryotes and eukaryotes. The biogenesis of a miRNA is begun from the transcription of the miRNA gene by RNA polymerase II, to a primary miRNA (pri-miRNA) containing hundreds of nucleotides length of RNA with a stem loop structure. The pri-miRNA is then cleaved by Drosha enzyme from the nonloop end to form the microRNA precursor (pre-miRNA), a double-stranded hairpin structure of RNA with 60–70 bp in length [142]. The pre-miRNA can be further processed into a matured miRNA by the RNaseIII Dicer and ultimately form a RNA-induced silencing complex (RISC) after it is exported to the cytoplasm by Exportin-5 [143]. RISC can functionally inhibit gene expression by binding to the 3′-untranslated region (3′-UTR) in a target mRNA, which is degraded if the miRNA:mRNA complex complementarity is perfect, or the translation is suppressed if the complementarity is not perfect. A compelling body of studies has evidenced that miRNAs play a critical role in the regulation of host genome expression at the posttranscriptional level. Emerging evidence has shown that aberrant miRNAs influence a wide range of biological processes including immune cell lineage commitment, differentiation, maturation, immune homeostasis, and normal function, by targeting various signaling pathways [144].

Recently, an increasing number of studies demonstrate that miRNA expression is related to various diseases including cancer, inflammatory, and autoimmune diseases [145]. Immunologically, miRNAs can regulate the development and function of immune cells; both a malfunctioned miRNA biogenesis machinery and a dysregulated expression of miRNAs may lead to an abnormal development and differentiation of immune cells and particularly a reduced suppressive function of regulatory T cells, resulting in systemic autoimmune diseases, such as SLE. Mechanistically, miRNAs may contribute to the development of various autoimmune diseases by targeting key components of signaling cascade in a variety of signaling pathways, including the Wnt pathways [146]. Indeed, there are several lines of evidence which have demonstrated immunoregulatory roles of miRNAs in the pathogenesis of autoimmune diseases through a mechanism by either directly or indirectly targeting Wnt signaling components (Table 2) [35, 72, 76, 78, 147]. There is accumulating evidence which revealed an important role of miRNAs in SLE pathogenesis by targeting Wnt signaling, and several independent microarray analyses have shown a significant difference of miRNA profiling between SLE patients and healthy controls. For example, a microRNA profiling analysis of peripheral blood mononuclear cells (PBMCs) of SLE patients identified 29 miRNAs that were downregulated in PBMCs of SLE patients as compared to healthy controls [148]. Pathway analysis further predicted that these miRNAs mainly targeted signaling pathways involved in diverse signaling transduction pathways, including the Wnt and mitogen-activated protein kinase (MAPK) signaling pathways [148]. Consistently, late microRNA profiling study of renal biopsies in human LN identified 24 miRNAs which were dysregulated (9 upregulated, 15 downregulated) relative to control renal tissues. Their predicted gene targets of these miRNAs included pathways associated with Wnt/*β*-catenin, TGF-*β*, NF-*κ*B, hepatocyte nuclear factor 4-alpha (HNF4A), and STAT3. In addition, the kallikrein-related peptidase 4 (KLK4) was further identified as a target of miR-422a, an upregulated miRNA in LN tissues [72].

The production of autoantibodies by uncontrolled hyperactivated B cells is a hallmark of SLE, and the early B cell factor 1 (EBF1) has been shown to play a key role in the development, activation, and proliferation of B cells through the AKT signaling pathway [149], which has recently been identified as a target of miR-1246 [150]. The miR-1246 thus may be a novel biological target in SLE treatment [150].

RA is another autoimmune and progressive systemic disease with high incidence. Recent reports show that altered expression and function of miRNAs might play an important role in the regulation of inflammatory innate immune responses in RA, which implied therapeutic potentials of miRNAs for RA. For instance, more abundance of miR-155 transcripts was found in PMBCs of RA patients compared with normal subjects. In addition, FLS exposed to TNF-*α* showed an upregulated expression of miR-155 and this miRNA is constitutively more highly expressed in RA FLS than in synovial fibroblasts of osteoarthritis (OA) patients. Functionally, the elevated expression of miR-155 suppressed the expression of MMP-3 and MMP-1 induced by cytokines and Toll-like receptor ligands, suggesting that miR-155 might have an important role in modulation of destructive behavior of RA FLS [151]. The miR-146a was another example, which also displayed a potential therapeutic effect for RA by inhibiting the expression of osteoclastogenesis.* In vivo* administration of miR-146a mimics by intravenous injection led to the suppression of cartilage and bone destruction [147].

Recently, a series of investigations conducted by Miao et al. demonstrated that several miRNAs, including miR-152, miR-375, and miR-663, were involved in the pathogenesis of RA by targeting Wnt signaling pathways [35, 74, 77, 78]. Among these, the miR-152 and miR-375 were downregulated, and the miR-663 was upregulated in RA patients or rat models. Mechanistically, enforced expression of miRNAs could indirectly upregulate the SFRP4 expression by targeting the DNMT1 in FLS, sequentially led to an inhibition of the canonical Wnt signaling, and results in a significant decrease of FLS proliferation [77]. miR-152 was specifically downregulated in arthritic rat model, enforced expression of miR-152 in FLS which resulted in a significantly downregulated DNMT1 expression, which in turn indirectly upregulated the SFRP4 expression and sequentially inhibited the canonical Wnt pathway activation, leading to a remarked decrease of FLS proliferation [77]. Consistently, miR-375 was also downregulated in FLS of arthritis rat model; an increased expression of miR-375 inhibited the canonical Wnt pathway by directly targeting Fzd8. Functionally, the increased miR-375 reduced the pathogenesis of arthritis rat model, as indicated by decreased expression of disease markers, such as MMP3 and fibronectin. Interestingly, such effect of miR-375 could be blocked in the presence of an active form of *β*-catenin [78]. Using a similar approach, the author further revealed that miRNA-663 could activate the canonical Wnt signaling by directly targeting APC [74]. In this context, the expression of miR-663 was significantly upregulated and APC expression was decreased in synovium from RA patients compared with controls. An inhibition of APC expression could activate canonical Wnt signaling through accumulation of *β*-catenin in FLS. Conversely, increasing miR-663 expression induced the FLS proliferation and the expression MMP3 and fibronectin during disease development [74].

Apart from the RA and SLE, differential expression of miRNAs participating in Wnt signaling pathways was also reported in other ADs, such as CD and primary biliary cirrhosis (PBC). Capuano et al. quantified 365 human miRNAs expression in human small intestinal tissues, and 20% of the examined miRNAs exhibited differential expression between CD and control children. Bioinformatics analysis predicted the NOTCH1 and *β*-catenin signaling were putative targets of these miRNAs. Furthermore, the NOTCH signaling was identified as a target of miR-449a in CD [73]. Qin et al. examined the alteration of miRNA expression profiles in PBMCs of patients with PBC and validated 6 of the 17 differentially expressed miRNAs [152]. Bioinformatics analysis further showed that the potential target genes of these miRNAs were involved in cell proliferation, cell differentiation, apoptosis, and signal transduction through mechanisms by targeting signaling pathways including the Wnt pathway [152].

## 6. Concluding Remarks and Future Perspectives

The Wnt signaling pathway plays a fundamental role in the embryonic development, cell proliferation, differentiation, stem cell maintenance, and tissue homeostasis, including the immune system development and immune regulation. A large body of study shows that a dysregulated Wnt signaling may lead to malfunctions of immune system and develop autoimmune diseases. Equally noteworthy, accumulating evidences have suggested that miRNAs are important regulators in pathogenesis of diseases, including the autoimmune diseases, through a mechanism by targeting Wnt signaling molecules. Indeed, increasing lines of evidence have indicated involvements of miRNAs in pathogenesis of autoimmune diseases by dysregulating Wnt signaling pathways, despite the fact that the precise underlying mechanisms remain unclear.

Although the regulatory roles of both the Wnt signaling and miRNAs in the pathogenesis of autoimmune diseases are now recognized, mechanisms by which miRNAs target Wnt signaling pathways in the autoimmune diseases remain largely unknown. Particularly, the miRNA expression profiling analysis of many autoimmune diseases has been performed, and signaling networks involving the targets of these miRNAs have also been predicted using bioinformatics tools. However, these targets need to be validated experimentally and clinically. The knowledge on the sequential molecular mechanisms of miRNAs targeting Wnt signaling cascade in autoimmune diseases may thus provide new diagnostic/prognostic markers and therapeutic targets for developing novel strategies and/or agents for treatments of immune diseases, through targeting Wnt signaling pathways.

## Figures and Tables

**Table 1:** Aberrant expression of molecules associated with Wnt signaling activity in autoimmunity diseases.

ADs	Wnt signaling	Key signaling molecule	Models	Evaluated factor(s)	Effect/mechanism	Reference(s)
RA	Canonical pathway	SFRP4	Rat RA model	MeCP2	Downregulating *β*-catenin by activating SFRP4 in RA rats	[34]
		GSK3*β*	MSCs	TNF-*α*	Ameliorating inflammatory responses	[43]
		DKK1	MSCs	NF-*κ*B	Suppressing Wnt signaling by upregulating DKK1; inhibiting inflammatory responses, and promoting bone resorption and formation	[44, 45]
		Wnt1, TCF/LEF1, SPRP1	FLS	proMMP3; fibronectin	An enhanced Wnt signaling promotes RA progression	[18]
		Wnt1; WISP3	FLS; synovial tissue	Inflammatory cytokines	High levels of Wnt1, Fzds, and WISP3 in RA tissues	[46]
	PKC mediated noncanonical pathway	Wnt5a/Fzd5	FLS	IL6; IL15; RANKL; NF-*κ*B	Contributing to the activated state of FLS in RA	[47]
	Wnt signaling	Wnt7b	Cartilage, bone, and synovium in RA and OA samples	TNF-*α* IL-1b; IL-6	Evidence of an involvement of Wnt signaling in the pathobiology of both RA and OA	[48]
		Wnt10b	Mouse	CD28 T cells	Inhibition of CD28 costimulation by CTLA-4Ig promotes T cell Wnt10b production and bone formation	[49]

AS	Wnt signaling	DKK1	Jurkat T cells	TNF-*α*	Downregulating Wnt signaling by increasing DDK1 expression and ameliorating inflammatory responses	[50]
		Wnt signaling	Chondrocytes	Induces differentiation of mesenchymal cells into osteoblast lineages	Active Wnt signaling contributes to osteophyte formation and joint remodeling	[7]
	Noncanonical pathway	Wnt5a, Wnt10b	MSCs	TNF-*α*	TNF-*α* induced Wnt5a and Wnt10b may be involved in the effects of inflammation on bone formation	[51]

SSc	Wnt signaling	Wnt2, Wnt3a, Wnt5a, Wnt10b, DDK2, LEF-1, WIF-1, *β*-catenin	Skin biopsies and peripheral blood samples from SSc patients and mouse model	TGF-*β*; IL4; IL13; IL17; IL33; IFN; IL13	An enhanced Wnt signaling promotes disease progression	[19, 52, 53]
		Wnt1, Wnt10b and DKK1	B cells, mouse model	IL6	The activation of Wnt signaling or inhibition of DKK1 induces severe fibrosis and lipoatrophy in animal models	[54]
		WIF-1, *β*-catenin	Fibroblasts from SSc	ATF3; HDAC3	An oxidative DNA damage induced by SSc autoreactive antibodies enables Wnt activation that contributes to fibrosis	[55]

AA	Canonical pathway	GSK3*β*, Wnt10b	NK cells	IFN-*γ*	A decreased Wnt signaling impairs anagen initiation and the ability of stem cells to drive differentiation of hair keratinocytes	[56]
	Wnt signaling	*β*-catenin, Wnt7a	C3H/HeJ mouse model of AA	Wnt signaling	Vitamin A enhances Wnt signaling to activate hair follicle SCs	[57]

CCL	Noncanonical pathway	Wnt5a, ROR1	HEK293 cells, leukemia B-1 cells	NF-*κ*B	ROR1 promotes CLL cells to receive survival signals	[58]
	Canonical pathway	Wnt/*β*-catenin	Leukemia B-1 cells, MEC-1 cells, CCL clinical samples	IL-6, inflammatory factors	Quercetin or metadherin inhibits leukemia cell expansion by blocking Wnt/*β*-catenin pathway and diminishes production of inflammatory factors in ADs and neoplasia	[59–61]

CIA	Wnt signaling	Fzd2	PGRN-deficient Tregs	TNF-*α*	Wnt signaling contributes to the PGRN regulation of Tregs	[62]

EAU	Canonical pathway	DKK3, SFRP2	RGM	IL17	Wnt inhibitors DKK3 and SFRP2 are downregulated in EAU; an enhanced Wnt signaling is involved in ERU pathogenesis	[6, 63]

IBD	Canonical pathway	TCF4	CD patients	Polymorphism analysis	Correlation of a functional variant TCF6 with early onset ileal CD	[64]
		TCF1, LRP6	Paneth cells	Alpha-defensins HD-5 and HD-6	A TCF-1-mediated Wnt signaling may contribute to the barrier dysfunction in CD	[65]
		LRP6	CD patients	Polymorphism analysis	Correlation of a functional variant LRP6 with early onset ileal CD	[33]

JRA	Canonical pathway	Wnt3a, WISP3, TCF1	SFMCs, Treg cells	FOXP3	A dysregulated Wnt signaling in the synovium inhibits Treg cell function and promotes JIA pathogenesis	[42]

MS	Wnt signaling	Wnt3a, Wnt5a, ROR2, *β*-catenin	EAE mice	Mechanical hyperalgesia and allodynia of paws in EAE mice	An aberrant activation of Wnt signaling contributes to the development of EAE-related chronic pain	[12]

Psoriasis	Canonical pathway	Activates Wnt signaling by LiCl	Manic-depressive patients	Thyroid microsomal antibodies	LiCl induced thyroid dysfunction and abrogated Treg cells suppressive capacity	[66]
	Wnt signaling	Wnt5a, Fzd2, Fzd5, DKKs, WIF-1	Biopsies of psoriasis patients	IL-1*α*, TNF-*α*, IFN-*γ*, TGF-*α*	Canonical Wnt signaling toward noncanonical pathways driven by interactions between Wnt5a and its cognate receptors in psoriasis, accompanied by impaired homeostatic inhibition of Wnt signaling by WIF-1 and dickkopf	[67]

SLE	Canonical pathway	DKK1	BM-MSCs	TNF-*α*	Wnt signaling plays a critical role in the senescence of SLE BM-MSCs through the p53/p21 pathway	[68]

T1DM	Canonical pathway	GSK3*β*	INS-1E rat insulinoma cells, rat islets	Cell proliferation and survival	An enhanced Wnt signaling by inhibiting CSK3 promotes *β*-cell proliferation	[69, 70]

UIP	Noncanonical pathway	Wnt5a	Primary fibroblasts of lung tissues with UIP	Fibronectin, *α*5-integrin, *β*-catenin	Wnt5a promotes fibroblast proliferation in IPF and UIP	[71]

AA: alopecia areata; ATF3: activating transcription factor 3; AS: ankylosing spondylitis; BM-MSCT: bone marrow-mesenchymal stem cell transplantation; CD: Crohn's disease; CIA: collagen-induced arthritis; CLL: chronic lymphocytic leukemia; CTL: cytotoxic T lymphocytes; EAE: experimental autoimmune encephalomyelitis; EAU: experimental autoimmune uveitis; ERU: equine recurrent uveitis; FLS: fibroblast-like synoviocytes; HDAC3: histone deacetylase 3; IPF: idiopathic pulmonary fibrosis; JRA: juvenile idiopathic arthritis; MEC: mucoepidermoid carcinoma cell; MeCP2: methyl-CpG-binding protein 2; MS: multiple sclerosis; MSCs: mesenchymal stem cells; NF-*κ*B: nuclear factor-kappa B; PBMCs: peripheral blood mononuclear cells; PGRN: progranulin; RA: rheumatoid arthritis; RANKL: receptor activator of nuclear factor kappa B ligand; RMG: retinal Müller glial cells; SCDH: spinal cord dorsal horn; SFMCs: synovial fluid mononuclear cells; SFRP4: frizzled-related protein 4; SLE: systemic lupus erythematosus; SSc: systemic sclerosis; TGF-*β*: transforming growth factor *β*; T1DM: type 1 diabetes mellitus; TNF-*α*: tumor necrosis factor alpha; WISP3: Wnt1-inducible signaling pathway protein 3; UIP: usual interstitial pneumonia.

**Table 2:** MicroRNAs target Wnt signaling in autoimmune diseases.

MicroRNA	Expression	Potential target of Wnt component(s)	Involvement of autoimmune diseases	Reference(s)
miR-422a	Upregulated	Wnt signaling and KLK4	Human LN	[72]

miR-449a	Upregulated	Wnt and NOTCH signaling	Human celiac disease (CD)	[73]

miR-663	Upregulated	Directly targeting APC of Wnt signaling	Downregulating APC to activate Wnt signaling and increase the FLS proliferation and the expression MMP3 and fibronectin in human RA	[74]

miR-26b	Downregulated	Wnt/GSK-3*β*/*β*-catenin pathway	Alleviating inflammation associated with RA by targeting Wnt signaling	[75]

miR-29a	Downregulated	Directly targeting DKK1 and GSK3*β* of Wnt signaling	Regulating TNF-*α* mediated bone loss in human AS	[76]

miR-152	Downregulated	Indirectly regulating SFRP4 by targeting DNMT1 and MeCP2	Pathogenesis of RA	[35, 77]

miR-375	Downregulated	FZD8	Arthritis synovial fibroblasts of rat AIA model	[78]

AIA: adjuvant-induced arthritis; APC: adenomatous polyposis coli; AS: Ankylosing spondylitis; CD: celiac disease; DNMT: DNA methyltransferase; FLS: fibroblast-like synoviocytes; FZD8: frizzled 8; KLK4: kallikrein-related peptidase 4; MeCP2: methyl CpG binding protein 2; MMP: matrix metalloprotease; RA: rheumatoid arthritis; SFRP4: secreted frizzled-related protein 4; TNF-*α*: tumor necrosis factor-alpha.

## Abbreviations

AA: Alopecia areata
AP1: Activator protein 1
APC: Adenomatous polyposis coli
AS: Ankylosing spondylitis
ATF3: Activating transcription factor 3
BM-MSCT: Bone marrow-mesenchymal stem cell transplantation
CAMK2A: Calcium/calmodulin-dependent protein kinase II alpha
CD: Crohn's disease
CIA: Collagen-induced arthritis
CLL: Chronic lymphocytic leukemia
CSNK1A1: Casein kinase 1
CTL: Cytotoxic T lymphocytes
Dvl1: Disheveled, dsh homolog 1
EAE: Experimental autoimmune encephalomyelitis
EAU: Experimental autoimmune uveitis
ERU: Equine recurrent uveitis
FLS: Fibroblast-like synoviocytes
GSK-3*β*: Glycogen synthase kinase 3 beta
HDAC3: Histone deacetylase 3
ILs: Interleukins
IPF: Idiopathic pulmonary fibrosis
JRA: Juvenile idiopathic arthritis
MEC: Mucoepidermoid carcinoma cell
MeCP2: Methyl-CpG-binding protein 2
MS: Multiple sclerosis
LRP: Low-density lipoprotein receptor-related protein
MSCs: Mesenchymal stem cells
NF-*κ*B: Nuclear factor-kappa B
NFAT: Nuclear factor of activated T cells
PBMCs: Peripheral blood mononuclear cells
PGRN: Progranulin
PKC: Protein kinase C
RA: Rheumatoid arthritis
RANKL: Receptor activator of nuclear factor-kappa B ligand
RMG: Retinal Müller glial cells
RhoA: Ras homolog gene family, member A
SCDH: Spinal cord dorsal horn
SFMCs: Synovial fluid mononuclear cells
SFRP4: Frizzled-related protein 4
SLE: Systemic lupus erythematosus
SSc: Systemic sclerosis
TGF-*β*: Transforming growth factor *β*
T1DM: Type 1 diabetes mellitus
TNF-*α*: Tumor necrosis factor alpha
WISP3: Wnt1-inducible signaling pathway protein 3
UIP: Usual interstitial pneumonia.
